# Fast-Setting Calcium Silicate-Based Pulp Capping Cements—Integrated Antibacterial, Irritation and Cytocompatibility Assessment

**DOI:** 10.3390/ma16010450

**Published:** 2023-01-03

**Authors:** Gabriel Kato, Pedro Sousa Gomes, Karin Hermana Neppelenbroek, Cláudia Rodrigues, Maria Helena Fernandes, Liliana Grenho

**Affiliations:** 1 Laboratory for Bone Metabolism and Regeneration, Faculty of Dental Medicine, University of Porto, 4200-393 Porto, Portugal; 2 LAQV/Requimte, University of Porto, 4100-007 Porto, Portugal; 3 Department of Periodontics and Prosthodontics, Bauru School of Dentistry, University of São Paulo, Al. Octávio Pinheiro Brisola, 9-75, Bauru 17012-901, SP, Brazil

**Keywords:** fast-setting cements, Biodentine, TotalFill, Theracal, ProRoot MTA, antibacterial activity, irritation potential, cytocompatibility

## Abstract

Calcium silicate-based cements (CSCs) are endodontic materials widely used in vital pulp-capping approaches. Concerning the clinical application, the reduced set time and pre-mixed formulations are relevant characteristics during the operative management of pulpal exposure, aiming to optimise the work time and improve cross-infection/asepsis control. Additionally, clinical success seems to be greatly dependent on the biological performance of the materials that directly contact the living pulp. As such, this work approaches an integrative biological characterisation (i.e., antibacterial, irritation, and cytocompatibility assays) of three fast-setting CSCs—Biodentine^TM^, TotalFill^®^ BC RRM™ Fast Putty, and Theracal LC^®^. These cements, after setting for 24 h, presented the expected topography and elemental composition (assessed by scanning electron microscopy, coupled with EDS analysis), in accordance with the information of the manufacturer. The set cements displayed a significant and similar antibiofilm activity against *S. mutans*, in a direct contact assay. Twenty-four-hour eluates were not irritant in the standardised CAM assay, but elicited distinct dose- and time-dependent cytotoxicity profiles on fibroblastic cells—i.e., Biodentine was devoid of toxicity, TotalFill presented a slight dose-dependent initial toxicity that was easily overcome, and Theracal LC was deleterious at high concentrations. When compared to long-setting ProRoot MTA cement, which highlighted the pursued integrative approach, Biodentine presented a similar profile, but TotalFill and Theracal LC displayed a poorer performance regarding antibiofilm activity/cytocompatibility features, and Theracal LC suggested eventual safety concerns.

## 1. Introduction

The exposure of dental pulp could occur intraoperatively during an iatrogenic procedure associated with an overzealous tooth preparation or upon the carious removal of an affected/infected dentin. Instead of the irreversible and complete pulpal extirpation preconised by the pulpectomy therapeutic approach, a conservative procedure could be alternatively performed through the partial removal of the coronal pulp, followed by hemostasis and pulp capping, achieved with the application of a bioactive dental cement [1,2,3,4]. This procedure, Vital Pulp Therapy (VPT), attempts to maintain pulp vitality through a biologically based approach, inducing tissue regeneration/mineralisation over the wounded dentin–pulpal complex, and arresting the development of apical periodontitis [4]. Part of the clinical success relies on the biological properties [5,6] of the materials that are applied as pulp-capping cements [3,7,8], with reported success even in cases of irreversible pulpitis [2,4,9]. Hence, a pulp-capping biomaterial should have a low cytotoxicity to maintain the vitality of the remaining pulp tissue and also allow the leaching of the bioactive substances that induce dentin regeneration. Additionally, exposure to carious pathogens within the oral environment, such as *Streptococcus mutans,* further demands effective antibacterial activity from these materials [10].

Calcium hydroxide (Ca(OH)_2_) is the main component of a range of pulp-capping cements that intend to stimulate mineralisation with the formation of a dentine barrier and inhibit bacterial growth in the underlying tissues, due to the high pH level. Nevertheless, calcium hydroxide has a low comprehensive strength and a low elastic modulus, which limit its independent application [11,12]. Therefore, Calcium Silicate-based Cements (CSCs), such as Mineral Trioxide Aggregate (MTA), have been developed, becoming clinical alternatives to the use of Ca(OH)_2_ alone [13,14,15] given their reduced solubility and yield of Ca(OH)_2_ after hydration [13,16,17].

A clinical limitation of MTA is its long setting time, requiring the application of a protective provisional restoration that is further replaced by a definitive restoration [18,19]. New formulations and ready-to-use mixtures of CSCs were used to attempt to enhance physical, biomechanical, and biological properties, as follows. Biodentine™ (Septodont, Saint-Maur-des-Fossés, France) consists of a CSC that is composed of tricalcium silicate, calcium carbonate, and zirconium dioxide, in the powder; whereas the liquid component is composed of water, with the addition of calcium chloride and a water-soluble polymer [20]. In this formulation, the setting time is reduced to around 10–12 minutes due to the addition of calcium chloride as an accelerator [21,22]. TotalFill^®^ BC RRM™ Fast Putty (FKG Dentaire SA, La Chaux-de-Fonds, Switzerland), on the other hand, consists of a premixed CSC with a shortened set time, near to 20 min, and is composed of monobasic calcium phosphate with tantalum pentoxide and zirconium [23]. Interestingly, alternative light-polymerised CSC formulations were also developed, which aimed for improved intraoperative control of the material’s setting. In this frame, Theracal LC^®^ (Bisco Inc., Schaumburg, IL, USA) is an available light-cure resin-modified tricalcium silicate dispersed in a hydrophilic monomer, composed of Bis-GMA and type I Portland cement, with the addition of barium sulfate as a radiopaque agent [24,25].

Of the reported endodontic cements, ProRoot MTA and Biodentine^TM^ are reliable pulp-capping CSCs able to induce dentin bridge formation and maintain pulp vitality after direct or indirect pulp-capping procedures [3,9,26]. On the other hand, resin-based pulp-capping agents were associated with a lack of complete hard bridge formation after application [6], and conflicting results related to the *in vitro* cytotoxicity/biocompatibility with Theracal LC^®^ were reported [9,10,11,12,13,14,15,16,17,18,19,20,21,22,23,24,25,26,27,28,29,30,31].

Calcium silicate-based cements exhibit distinct cytotoxic profiles within eukaryotic cells, due to diverse factors, i.e., composition, physico-chemical features, setting reaction, setting time, and the leaching kinetics of the unset and set cements. Adding to this complexity, the wide variety of experimental protocols that have been used regarding the cell line, type of assay (direct/indirect contact), exposure time, and assessment parameters, frequently lead to conflicting results on the establishment of the biological profile of these endodontic cements [9,32]. There are similar concerns regarding the available data on the antibacterial activity of CSCs [10,33]. Additionally, studies have usually addressed two issues—cytotoxicity and antibacterial activity—separately, hampering results comparisons and the establishment of safety/activity patterns in relation to biological outcomes. Studies directly comparing the integrated biological response to these cements are, thus, limited.

As such, as a highlight, the present work aims to provide an integrative view of the biological profile of elected CSCs, namely three currently used fast-setting CSCs—Biodentine^TM^, TotalFill^®^ BC RRM™ Fast Putty, and Theracal LC^®^. The set cements were assessed for topography and elemental composition. For the biological profile, the set cement was assessed for its activity against *S. mutans*—the major causative agent of dental caries (direct contact assay), and the cements’ eluates were tested for irritation potential (CAM assay) and cytotoxicity according to ISO 10993 [34]. The results were compared to those observed with ProRoot MTA, due to its established clinical success in spite of its long setting time, and Dycal, an early-generation calcium-based cement, selected as a “historical” control, which was once recognised as the gold standard for pulp-capping applications, with more than 50 years of clinical application [3,9].

## 2. Material and Methods

### 2.1. Calcium Silicate-Based Cements

The present study evaluated the following dental materials, Biodentine™ (Septodont, Saint-Maur-des-Fossés, France), TotalFill^®^ BC RRM™ Fast Putty (FKG Dentaire SA, La Chaux-de-Fonds, Switzerland), and Theracal LC^®^ (BISCO Inc., Schaumburg, IL, USA), as fast-setting CSCs for pulp capping; ProRoot^®^ MTA (Dentsply Maillefer, Ballaigues, Switzerland) and Dycal^®^ (Dentsply DeTrey GmbH, Dresden, Germany) were included as control materials. The chemical compositions, as provided by the manufacturer and batch number, are detailed in Table 1.

*Set cements*. Dental cements were manipulated, in accordance with the instructions of the manufacturer, under aseptic conditions. The cement samples were established with 1 mm thickness on a plastic tissue culture coverslip (∅ 13 mm, 1.9 cm^2^, Sarstedt Inc., Newton, NC, USA) and allowed to set overnight at 37 °C in a 100% humidified atmosphere for further characterisation.

*Cement extracts*. The extracts were prepared according to ISO 10993 part 12 guidelines [35]. Briefly, set cements (1 mm thickness circles with ∅ 13 mm, 1.33 cm^2^) were placed into sterilised 24-well plates and incubated in cell culture medium (0.6 mL/well), i.e., α-minimum essential medium (α-MEM) supplemented with 10% (*v*/*v*) fetal bovine serum, 100 IU/mL penicillin, 100 µg/mL streptomycin, and 2.5 µg/mL amphotericin B (all from Gibco^®^, Waltham, MA, USA), for 24 h at 37 °C in a humidified atmosphere (5% CO_2_/air). After incubation, the media, hereinafter extracts, were collected, filtered (0.2 μm), and diluted in cell culture medium, i.e., 50%, 25%, 15%, 10%, and 1%. The undiluted extracts showed pH values of ~9.5. For the diluted extracts, the buffering capacity of the culture medium kept the pH ~7.4 (the intracellular pH).

### 2.2. Topography and Elemental Analysis of the Set Cements

Material characterisation was performed by scanning electron microscopy (SEM) and energy dispersive spectroscopy (EDS). Briefly, CSCs, previously established on coverslips and set overnight, were coated with an Au/Pd thin film (SPI Module Sputter Coater, West Chester, PA, USA) and subjected to high-resolution SEM with X-ray microanalysis in backscatter mode (SEM-BSE), followed by EDS analysis (FEI Quanta 400 FEG ESEM/EDAX Genesis X4M; Fei Company, Hillsboro, OR, USA). Semi-quantitative elemental composition of each sample was performed for the viewing area (5 random positions per sample) as well as for point locations (3 analyses for each area).

### 2.3. In Vitro Prevention of Antibiofilm Formation by the Set Cements

The prevention of the biofilm formation by CSCs was inferred by the characterisation of sessile population using quantitative viable counts and SEM visualisation. 

A standard bacterial suspension of *Streptococcus mutans* DSM 20523 (DSMZ, Braunschweig, Germany) at a density of 10^8^ cells/mL, in brain heart infusion (BHI; Liofilchem, Roseto degli Abruzzi, Italy), was seeded over CSC samples (established on coverslips, 1.9 cm^2^, and set overnight, as described above) and then incubated for 24 h at 37 °C and 120 rpm. Coverslips (1.9 cm^2^) without cement were used as control. After incubation, the samples were carefully washed with sterile saline solution to remove loosely attached bacteria. For viable counts, samples were transferred to tubes with sterile saline solution and sonicated for 10 min (Sonorex; Bandelin, Berlin, Germany) to dislodge sessile bacteria. The suspensions were serially diluted and inoculated in BHI agar plates. After 24 h incubation at 37 °C, colony forming units (CFUs)/mL were quantified. Results were expressed as mean Log10 CFU/mL. CFU data were transformed to logarithmic scale only after statistical analysis of the raw data.

For SEM visualisation, pre-washed samples were fixed with 1.5% glutaraldehyde in cacodylate for 30 min. The samples were then dehydrated in sequentially graded ethanol solutions (50% to 100%) and critical-point-dried (CPD 7501, Polaron Range). Finally, the samples were coated with an Au/Pd thin film (SPI Module; West Chester, PA, USA) and visualised by SEM (FEI Quanta 400 FEG ESEM/EDAX Genesis X4M; Fei Company, Hillsboro, OR, USA).

### 2.4. In Vivo Irritation Potential of the Undiluted Extracts

The irritation potential of the CSC extracts was tested using the *in vivo* chorioallantoic membrane (CAM) assay according to the Interagency Coordinating Committee on the Validation of Alternative Methods (ICCVAM) guidelines [36]. Accordingly, fertilised chicken eggs were incubated with hourly scheduled rotation at 37 °C in a 60% humidified atmosphere in an Octagon Advance incubator (Brinsea Products, Inc., Weston-super-Mare, UK). On day 9 after fertilisation, an eggshell window was created to access the CAM beneath. Subsequently, the undiluted cement extracts (prepared as described above) were loaded onto silicon O-rings randomly placed on the CAM to retain the sample and delimit the area of analysis. For a contact period of up to 5 min, the CAM was imaged for signs of irritation using a stereomicroscope (Stemi 305, Zeiss, Oberkochen, Germany) and an attached imaging system (Axiocam 208 color, Zeiss, Oberkochen, Germany).

The irritation potential was scored by the occurrence of specific damages to membranes and/or vessels, regarding haemorrhage, clotting, and vascular lysis, which were interpreted in comparison to a negative (0.9% NaCl) and positive (1% SDS) control. Irritation index was assigned semi-quantitatively using a grading system according to the Luepke method, from 0 (no reaction) to 3 (strong reaction) [36]. Each test was carried out in quintuplicate. 

### 2.5. In Vitro Cytocompatibility of the Extracts

The cytocompatibility of CSCs was tested by an indirect contact assay according to the standard cytotoxicity assessment established by the International Organization for Standardization [34]. L929 mouse fibroblast cells (NCTC clone 929, ATCC) were seeded at a density of 10^4^ cells/well into 96-well plates and incubated in culture medium (composition as described above) for 24 h at 37 °C, in a humidified atmosphere (5% CO_2_/air). After incubation, adhered cells were exposed to undiluted (100%) and diluted (50% to 1% dilutions) extracts for periods up to 3 days, during which the medium remained unchanged. Cell culture medium was used as control. The plates were further maintained under standard incubation conditions.

Cell viability and cell metabolic activity were evaluated by live/dead staining and MTT assay, respectively. For live/dead staining, cultures exposed for 24 h to the extracts were incubated with 1 µM Calcein AM (BioLegend^®^, San Diego, CA, USA) and 50 µL/mL of propidium iodide (PI; BD Biosciences^®^, San Jose, CA, USA) solution for 15 min at 37 °C, protected from light. Fluorescent cells were further recorded using Celena S Digital Imaging System (Logos Biosystems^®^, Gyeonggi-do, South Korea). For MTT assay, measuring mitochondrial dehydrogenase activity, cell cultures exposed to the extracts for 24, 48, and 72 h were incubated with 10% of 3-(4,5-dimethylthiazol-2-yl)-2,5-diphenyltetrazolium (MTT, 1 mg/mL, Sigma-Aldrich, St. Louis, MO, USA) for 3 h at 37 °C. The formed formazan precipitates were solubilised in dimethyl sulfoxide (DMSO, Sigma-Aldrich, St. Louis, MO, USA) for 15 min, and absorbance was measured at 550 nm on a microplate reader (Synergy HT, Biotek^®^, Santa Clara, CA, USA). Results were presented as percentage (%) of control (cultures performed in cell culture medium). According to the ISO 10993-5 guidelines [37], which describe methods to assess the *in vitro* cytotoxicity of medical devices, 70% viability of the control is considered the “cut-off” value for the device to be categorised as non-cytotoxic.

### 2.6. Statistical Analysis

Results are presented as mean ± standard deviation of three independent experiments, with three replications for each experiment, unless otherwise indicated. Statistical analysis was performed with IBSS^®^ SPSS Statistics package (v. 28.0, IBM, Armonk, NY, USA), and graphics were constructed with GraphPad Prism 8.0.1 (GraphPad Software, Inc.; San Diego, CA, USA). Comparison of the experimental conditions was performed by the *t*-test and the comparison of several groups by the one-way analysis of variance (ANOVA). The Shapiro–Wilk test was used for the assessment of the normality of data. Statistically significant differences were considered at *p*-values ≤ 0.05.

## 3. Results 

### 3.1. Topography and Elemental Composition of the Set Cements

**Figure 1:**
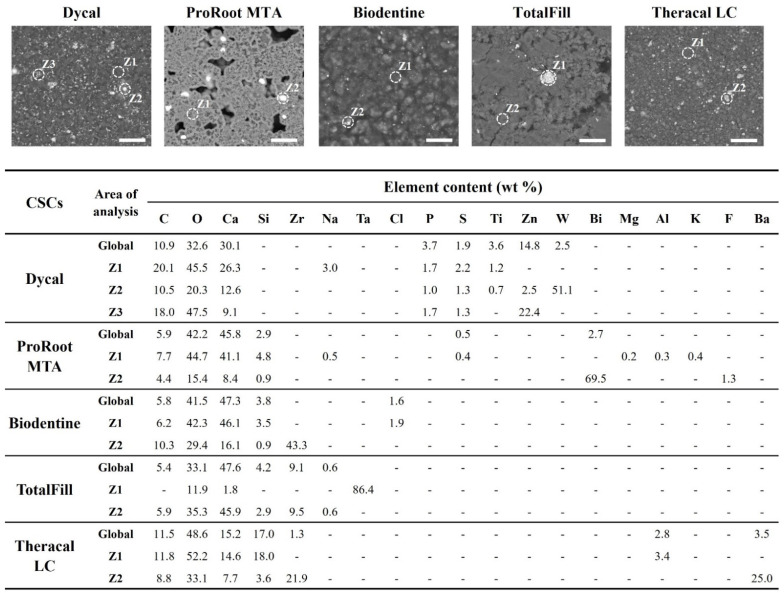
Back-scattered scanning electron micrographs of the calcium silicate-based cements (CSCs) showing microstructural components (scale bar 10 µm) and respective elemental composition evaluated by EDS, for the view area and for the pointed zones in the images.

SEM images of the CSCs’ set samples revealed relatively different and heterogeneous surfaces with the punctual presence of lightened areas, after back-scattered electron (BSE) imaging (Figure 1). Global elemental analysis by semi-quantitative EDS revealed carbon (C), oxygen (O), and calcium (Ca) in all the assayed cements. Silicon (Si) was also identified in all cements, except Dycal. The latter was the only cement to present phosphorus (P) and titanium (Ti) in its microstructure as well as clear aggregates of zinc (Zn) and tungsten (W) (Figure 1, Dycal Z2 and Z3). ProRoot MTA presented traces of magnesium (Mg), aluminium (Al), potassium (K), and fluorine (F) as well as bright aggregates of bismuth (Bi) (Figure 1, ProRoot MTA Z2). Small aggregates of zirconium (Zr) were detected in the Biodentine and Theracal LC samples (Figure 1, Z2 of the respective cement). Zr was also identified in the TotalFill samples but was imbued in the matrix (Figure 1, TotalFill Global and Z2). This cement also presented large aggregates of tantalum (Ta) (Figure 1, TotalFill Z1). Lastly, Theracal LC showed traces of Al and accumulations of barium (Ba) and Zr (Figure 1, Theracal LC Z1 and Z2, respectively).

### 3.2. Prevention of Antibiofilm Formation

**Figure 2:**
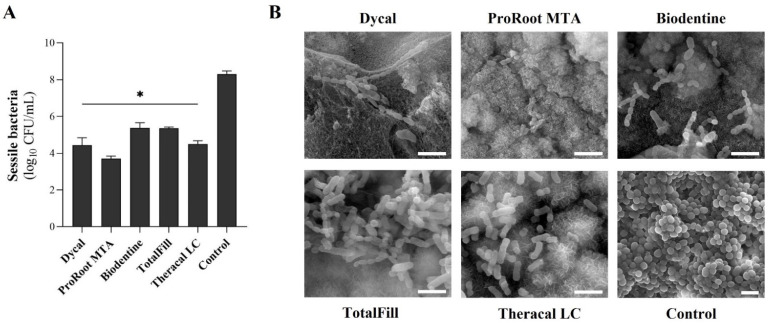
(**A**) Viable counts of the sessile *S. mutans* population after a 24 h direct incubation with the set CSCs, expressed as mean Log_10_ CFU/mL. (**B**) SEM images of *S. mutans* morphology on the surface of the materials (scale bar 2 µm). * Statistically significant difference from the control, *p* ≤ 0.05.

The data showed that the set cements caused a strong and significant reduction in the sessile population of *S. mutans*, as compared to the control (Figure 2A). Specifically, ProRoot MTA showed a 4.6 log reduction in the sessile population, followed by Dycal and Theracal LC with reductions of 3.9 and 3.8 logs, respectively. Biodentine and TotalFill presented a 2.9 log reduction. Comparatively, no significant differences were attained between the assayed cements. These results were further confirmed by SEM analysis. Representative images showed a small number of aggregates of bacteria scattered along the surface of the endodontic cements, with an atypical elongated morphology and a similar organisation throughout the distinct cement formulations. In contrast, the control samples were heavily colonised by *S. mutans*, with its characteristic sphere-shaped morphology (Figure 2B).

### 3.3. In Vivo Irritation Potential 

**Figure 3:**
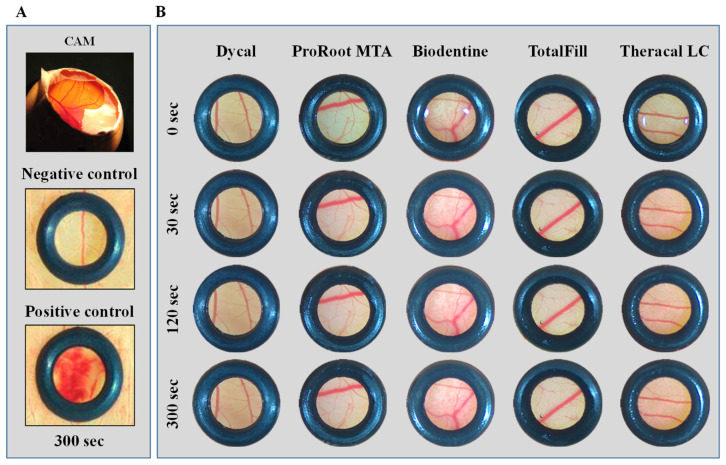
*In vivo* HET-CAM irritation assay. (**A**) Representative images of CAM and the negative and positive controls after exposures of 300 s. (**B**) Representative CAM images after exposure to the undiluted cement extracts for periods up to 300 s. Microscopic images were taken at 10× magnification.

Undiluted cement extracts (100% CSCs’ extract concentrations) were evaluated for the irritation potential by the CAM assay. All cements behaved similarly. In the representative CAM images (Figure 3), qualitative specific reactions related to hyperaemia, haemorrhage, vascular lysis, and coagulation were not observed after direct contact with the pure extract for periods up to 300 s according to the guideline. The CAM appearance was similar to that of the negative control but substantially different from the positive control. The irritation index according to the Luepke method was 0 (no reaction) for all the experimental conditions and the negative control [36].

### 3.4. Cytotoxicity to Eukaryotic Cells

Adherent L929 fibroblasts were exposed to the undiluted (100%) and diluted (50% to 1%) cement extracts for periods of 24 h to assess initial toxicity and for 48 and 72 h to disclose the effect on cell proliferation.

**Figure 4:**
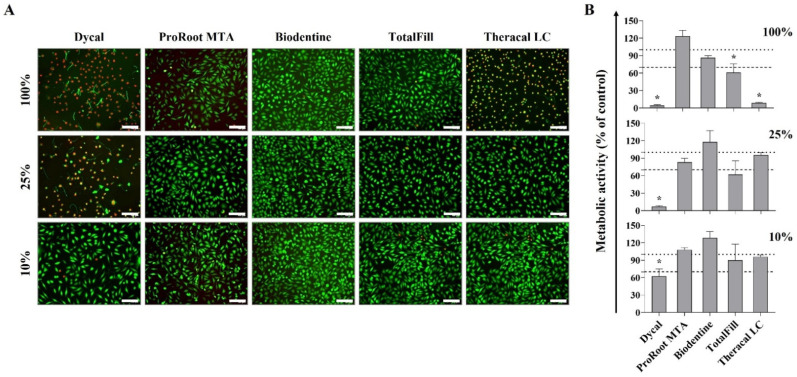
(**A**) Live/dead assay fluorescence images of L929 fibroblasts exposed to 100%, 25%, and 10% cement extracts for 24 h; live cells (green) and dead cells (red) (scale bar 200 μm). (**B**) Metabolic activity measured in the same cultures, expressed at percentage of control, set at 100% (dotted line); the “cut-off” value of 70% of the control is also shown (dashed line). * Statistically significant difference from the control, *p* ≤ 0.05.

After 24 h exposure, fluorescence images of the live/dead assay were acquired, and representative micrographs are depicted in Figure 4A, for the cells exposed to 100% extracts and to 25% and 10% extract dilutions. Cell viability was affected by the cement and/or the extract dilution. For Dycal, the undiluted and the 25% dilution extract caused significant cell death, with only a few viable cells visible; however, exposure to the 10% dilution showed a very low number of dead cells. The undiluted Theracal LC extract also presented a high initial toxicity (though lower than that of Dycal), which was not observed following exposure to the diluted extracts. The extracts from the other cements (i.e., ProRoot MTA, Biodentine, and TotalFill), either undiluted or diluted, did not show signs of initial toxicity in the live/dead assay. The MTT assay performed on the 24 h exposed cultures (Figure 4B), assessing the cell metabolic activity, revealed a similar trend to that reported on the live/dead assay.

**Figure 5:**
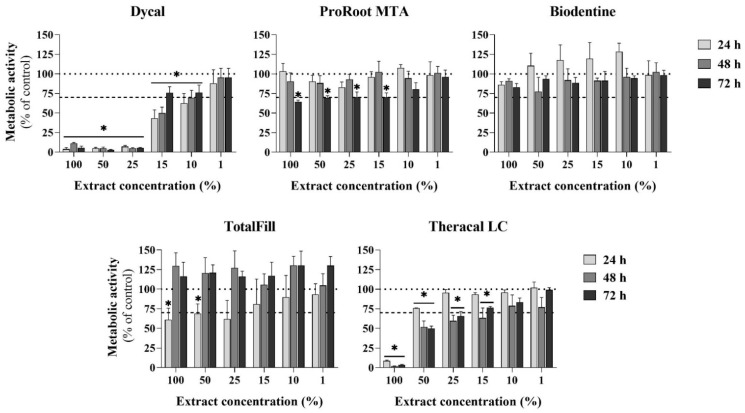
Cell proliferation of L929 fibroblasts exposed to undiluted (100%) and diluted (50%, 25%, 15%, 10%, and 1%) cement extracts for 24, 48, and 72 h. Results are expressed as percentage of control, set at 100% (dotted line); the “cut-off” value of 70% of the control is also shown (dashed line). * Statistically significant difference from the control, *p* ≤ 0.05.

The metabolic activity of the cultures was evaluated throughout a 72 h exposure to the extracts (Figure 5), allowing for the disclosure of cell viability and proliferation.

Cells hardly proliferated in the presence of concentrated Dycal extracts (100% to 25%), and cell growth was dose-dependent for lower extract concentrations (15% to 1%). The values were around 70% of the control for the 10% extract and were similar to the control for the 1% extract. For each extract dilution, the cell proliferation increased with the culture time (24 to 72 h; this was particularly evident for the 15% extract). 

ProRoot MTA (either undiluted or upon dilution) did not disturb cell proliferation for exposures of 24 and 48 h, but the values decreased at 72 h (~70% of the control; within 100% to 15% dilution range).

With the Biodentine extracts, the cell growth was not affected, and, further, increased values were observed upon exposure for 24 h for the 50–10% extracts (though without significant differences). 

TotalFill presented some initial toxicity (24 h exposure) for the range of the 100% to 25% extracts, but the cultures fully recovered to values similar to the control for longer exposures (48 and 72 h). However, across the concentration range tested, proliferation was never below the “cut-off” value of 70% of the control.

Concerning Theracal LC, cells were unable to proliferate in the presence of the undiluted extract, and the values were found to be lower than 70% regarding exposure to the 50% extract (48 and 72 h). Significantly reduced levels, as compared to the control, were further verified for the 25% and 15% extracts—at 48 and 72 h. In this range—50%, 25% and 15% extracts—toxicity increased with the exposure time, i.e., it was higher upon 48 and 72 h exposure (compared to that upon 24 h exposure). No significant differences were found in the lower dilution range of the Theracal LC extract, i.e., 10% and 1%, as compared to the control.

## 4. Discussion

Pulp-capping materials are placed directly over the exposed pulp, attempting to ensure pulp vitality and prevent the need for further endodontic treatment, within the VPT therapeutic approach. As such, materials should present low potential to be irritating, be cytotoxic, or induce other adverse biological outcomes. Antibacterial activity is also a key issue for the clinical success of this approach, due to the possibility of secondary infections caused by remnant bacteria or microleakage. 

ProRoot MTA has been extensively studied and, due to its proven biocompatibility and long-term track record of clinical success, is the most validated material for pulp capping [9,13]. However, due to its long setting time and difficult handling, other calcium silicate-based materials, which were introduced later and are faster-setting, are being used in direct pulp-capping [13]. Compared to those on ProRoot MTA, fewer studies are dedicated to these cements, and inconsistencies arise from the *in vitro*, *in vivo*, and clinical studies [9]. The present study addressed the biological profile of three fast-setting calcium silicate-based cements—Biodentine, TotalFill, and Theracal LC, for their irritating potential, antibiofilm activity, and cytocompatibility. For comparative purposes, ProRoot MTA was included. Dycal, an early-generation material, was also analysed as a “historical” control.

The CSCs set for 24 h were characterised using a combination of two techniques, namely SEM, which enabled the assessment of material microstructure, and EDS, which was used for semi-quantitative elemental analysis. Accordingly, the samples presented a heterogeneous surface where the presence of C, O, Ca, and Si, typical of calcium silicate-based cements, was detected (Figure 1), except for Dycal (a non-silicate-based cement). In Dycal, the presence of P, Ti, and small aggregates of Zn was also identified, which confirmed the results of Gong et al. [38]. ProRoot MTA presented traces of Mg and Al, indicating that the cement is a Portland type [39], and also K and F. On Theracal LC, traces of aluminium were also detected in the cement phase of the material, as reported by other authors [40]. As expected, all CSCs showed the presence of some sort of radiopacifier, as highlighted by the BSE-SEM micrographs (Figure 1), as follows. For instance, in Dycal, the presence of tungsten may be assigned to calcium tungstate (CaO_4_W) [38]; ProRoot MTA presented bright aggregates of bismuth, likely assigned to bismuth oxide [15], while Biodentine displayed zirconium linked to zirconium oxide [41]. TotalFill showed large amounts of tantalum assigned to tantalum oxide [39], and, on Theracal LC, barium and zirconium were detected and ascribed to barium zirconate [40,42], which provide radiopacity to these cements. 

Anticariogenic proprieties must be pondered, whereas it is an important issue both to inhibit caries progression in selective caries removal and to prevent the infection relapse in the marginal structure of a dental restoration. The antimicrobial properties of pulp-capping materials have been extensively tested in the literature, though with conflicting or contradictory results. This has been ascribed to a great heterogeneity in the methodologies used and a lack of standardisation, with different contact times, material handling, and evaluation parameters, and a diversity of microorganisms and culture conditions included in the studies [10,43]. Considering that most of the bacteria are in sessile growth in dental infections, the current study was devised to comparatively evaluate the cements’ capability to lessen bacterial adhesion and, consequently, biofilm formation when exposed to the oral environment. Therefore, the materials were incubated with *S. mutans*, in a direct contact test for 24 h, to simulate the contact of the microorganism with the cements, and to allow bacterial adhesion and biofilm formation. In the literature, there is a paucity of *in vitro* studies addressing CSCs’ effects on the prevention of biofilm formation [10,44,45], and even fewer studies include light-curable tricalcium silicate—Theracal LC [39,46]. Further, the agar diffusion test, used in most studies, only reflects the diffusion ability of the cement, not its direct antimicrobial potential, yielding frequently limited effects [47]. The direct contact test gives more reliable results [10]. 

In the present work, the results demonstrated that all CSCs tested, including the fast-setting ones, were able to significantly reduce *S. mutans* adhesion to the cements’ surface (Figure 2). Sequentially, ProRoot MTA presented the highest inhibition of *S. mutans* adhesion, followed by Dycal, Theracal LC, and, lastly, Biodentine and TotalFill, despite the absence of significant differences between the materials. For all samples, the pH of the bacterial suspension surrounding the set cement samples was around 9.5. Overall, the antimicrobial activity of calcium silicate-based cements has been attributed to their surface of contact and the release of hydroxyl ions in the aqueous environment with the associated alkalinisation [5,10,48,49,50,51]. Hydroxyl ions are able to react with various biomolecules, such as lipids and proteins, disrupting the microorganisms’ cell membranes, essential structures to bacterial cell survival [52]. This reactivity appears to be indiscriminate, and the free ions rarely diffuse away from their site of generation [51]; this is a most relevant issue, as the induced toxicity seemed to be restricted to the materials’ surface, not influencing the commensal oral microbiota or adjacent tissues. This favoured bacteria elimination when in close proximity to the material surface, minimising the chance of biofilm formation. In addition, the associated alkalinisation of the surrounding fluids [39,46,53,54], exerted a significant ecological pressure on *S. mutans* and other cariogenic microorganisms, as low-pH environments are crucial for their survival and pathogenicity [55,56]. Further, the composition of the cements and the crystalline phases may also account for the observed antimicrobial activity, namely due to the ionic release owing to the presence of metal oxides.

The safety profile of pulp-capping cements is a major concern. In the present work, it was analysed in 24 h extracts prepared according to ISO 10993-12 guidelines. Nonetheless, the biological response may differ regarding the assessment of freshly prepared or setting materials, which should be evaluated in future studies. For all set cements, the pure extract did not show any irritation potential, as determined by the HET-CAM irritation assay (Figure 3). Whether or not this assay seems to produce translational data regarding irritation outcomes, based on ICCVAM guidelines [36], it cannot fully disclose irritation potential in mammal models, particularly for long-term time points. 

Next, cytotoxicity to eukaryotic cells was analysed using an indirect assay involving the exposure of L929 fibroblasts to the undiluted (pH ~9.5) and diluted (pH ~7.4) extracts. Due to the cements’ individual compositions and the inclusion of distinct additives (reaction and physical modifiers), the set cements yielded extracts with different compositions, inducing distinct cellular responses [39,42]. The hydraulic calcium silicate-based cements, i.e., ProRoot MTA, Biodentine, and TotalFill, showed low or no toxicity (Figure 4 and Figure 5). The three materials have a water-based chemistry and set by reaction with water, forming calcium hydroxide as a by-product of the hydration reaction, which raises the pH of the surrounding environment [39]. In the present study, only the undiluted extracts showed an elevated pH (~9.5). For the diluted extracts, the buffering capacity of the culture medium kept the pH around 7.4 (the intracellular pH). Biodentine behaved similarly to the control. ProRoot MTA and TotalFill showed a slightly toxic effect at the higher extract concentrations, although with a different toxicity pattern, suggesting distinct toxicity mechanisms. For the ProRoot MTA extracts, the cell viability decreased following longer exposures (3 days); thus, a cumulative effect from the toxic compounds might be hypothesised, namely due to the presence of Bi (from the bismuth oxide radiopacifier) [57]. For the TotalFill extracts, toxicity was observed only initially (24 h exposure); thus, the stabilisation of the culture conditions, i.e., a progressive normalisation of the pH in the 5% CO_2_ incubation conditions, might contribute to the cells’ recovery [48]. Nevertheless, for this cement, within the tested dilution range and exposure periods, the cell viability was never less than 70% of the control, the “cut-off” value for a material to be considered safe [34]. The low cytotoxicity of these cements is in line with the available information, especially the proven cytocompatibility of ProRoot MTA and Biodentine [58]. Comparatively, the light-cured Theracal LC, a resin-modified calcium hydroxide fortified with calcium silicate, presented a higher cytotoxicity (Figure 4 and Figure 5). Cell proliferation was dose- and time-dependent and was observed only with concentrations ≤50%, and toxicity increased with the exposure time (48 and 72 h). Mostly, the published *in vitro* cytotoxicity data also showed the lower cytocompatibility of Theracal LC compared to ProRoot MTA and Biodentine [9,28,59]. Theracal LC toxicity was mainly attributed to resin monomers, which may remain unpolymerised upon setting [28]. Further, previous studies showed that cured Theracal LC released specific toxic additives, camphoroquinone and ethyl-4-(dimethylamino)benzoate [60], which, probably, as suggested in the present work, showed a cumulative toxic effect. Dycal, an early-generation calcium-based cement, showed the highest toxicity among the tested materials, with cell growth occurring only with extract concentrations ≤15% (Figure 4 and Figure 5). 

Overall, the results of the present study are similar to those reported recently by Manaspon et al. [61], who also performed a study with set cements’ extracts prepared according to the ISO 10993 protocol. They reported a lower toxicity for ProRoot MTA and Biodentine compared to that for Theracal LC and Dycal, including the comparatively higher toxicity of Dycal [61]. Nevertheless, it should be noted that the closed *in vitro* culture conditions are far from the dynamic *in vivo* context of the extracellular fluid flow and concentration gradient that would contribute to the progressively reduced levels of the leaching compounds neighbouring the cells/tissues, lowering cytotoxicity. Accordingly, the three cements are clinically used as pulp-capping materials [9,11,62,63,64,65]. Comparative studies with ProRoot MTA show some inconsistencies in the clinical outcomes, especially concerning Theracal LC, with an eventually unfavorable performance/safety profile being suggested by some studies [9,62,66]. The *in vitro* results reported in the present study follow the same trend regarding the possible higher cytotoxicity of Theracal LC compared with the other fast-setting cements. This suggests that *in vitro* studies, although with inherent limitations, may provide preliminary guiding information and clarify the underlying mechanisms, feeding more complex and integrative approaches to evaluate the cements’ biological profiles.

## 5. Conclusions

The fast-setting cements Biodentine, TotalFill, and Theracal LC, set for 24 h, presented the expected elemental composition, in accordance with the listed information of the manufacturer. The set cements displayed a significant and similar antibiofilm activity against *S. mutans* in a direct contact assay. Twenty-four-hour eluates were not irritant in the standardised CAM assay but elicited distinct dose- and time-dependent cytotoxicity profiles with fibroblastic populations—i.e., Biodentine was devoid of toxicity, TotalFill presented a slightly dose-dependent initial toxicity that was easily overcome, and Theracal LC was deleterious at high concentrations. Highlighting the pursued integrative approach, compared to the long-setting ProRoot MTA cement, Biodentine presented a similar profile, but TotalFill and Theracal LC displayed a inferior performance in antibiofilm activity and cytocompatibility, particularly Theracal LC.

## Figures and Tables

**Table 1:** Calcium silicate-based cements used and respective compositions, manufacturers, and batch numbers.

Material	Composition	Manufacturer	Batch No.
Dycal^®^	Base paste: titanium dioxide, barium sulphate, glycol disalicylate Catalyst paste: calcium hydroxide, zinc oxide, zinc stearate, ethyl toluene sulphonamide	Dentsply DeTrey GmbH, Dresden, Germany	00071192
ProRoot^®^ MTA	Tricalcium silicate, dicalcium silicate, tricalcium aluminate and calcium sulfate	Dentsply DeTrey GmbH, Dresden, Germany	0000301574
Biodentine^TM^	Powder: tricalcium silicate, dicalcium silicate, calcium carbonate, iron oxide, zirconium oxide Liquid: water with calcium chloride and soluble polymer (polycarboxylate)	Septodont, Saint-Maur-des-Fossés, France	B27532
TotalFill^®^ BC RRM^TM^ Fast Putty	Zirconium oxide, tantalum oxide, calcium silicate, calcium phosphate monobasic and fillers	FKG Dentaire SA, La Chaux-de-Fonds, Switzerland	2100004308
Theracal LC^®^	Portland cement, BisGMA, barium zirconate	Bisco, INC., Schaumburg, IL, USA	2100004308

## Data Availability

The data presented in this study are available on request from the corresponding author.
